# Gemini Amphiphiles: Robust Cytosolic Delivery of Therapeutic Biomacromolecules through Non-Endocytic Translocation Pathways

**DOI:** 10.1021/acscentsci.3c00793

**Published:** 2023-07-07

**Authors:** Yu Zhao, Douglas Wich, Qiaobing Xu

**Affiliations:** Department of Biomedical Engineering, Tufts University, Medford, Massachusetts 02155, United States

Delivery of therapeutic proteins into living cells is highly desirable in numerous biological, biotechnological, and therapeutic applications. However, these applications have not yet been fully realized because of the poor plasma membrane permeability of protein molecules, which results in limited intracellular exposure. Strategies using vectors are particularly attractive because they can be applied to facilitate protein transport across plasma membranes both *in vitro* and *in vivo*. These vectors typically gain access to the interior of the cells through endocytic pathways. However, endosomal escape is inefficient for many vectors. Specifically, most protein-loaded vectors that enter cells are entrapped in endosomes, resulting in only a small amount of protein entering the cytoplasm to function. Certain proteins can still trigger sufficient intracellular responses despite having a relatively low efficiency of cytosolic delivery. However, this issue still limits the application of proteins that require high intracellular concentrations for therapeutic activity. In this issue of *ACS Central Science,* Ping, Chen, Liu, and co-workers established a molecular library of gemini amphiphiles (GAs) with tunable charges and hydrophobic properties. The vehicles prepared by optimized GAs achieved robust cytosolic delivery of proteins with various molecular weights (MWs) and isoelectric points (pIs). According to the theory proposed in this article, an important reason for the highly efficient protein delivery is that the internalized mechanism is predominantly mediated via lipid raft-dependent membrane fusion, rather than the classical endocytic pathway. This means that proteins can be delivered directly to the cytoplasm using GA-based vehicles.^1^

**Figure 1:**
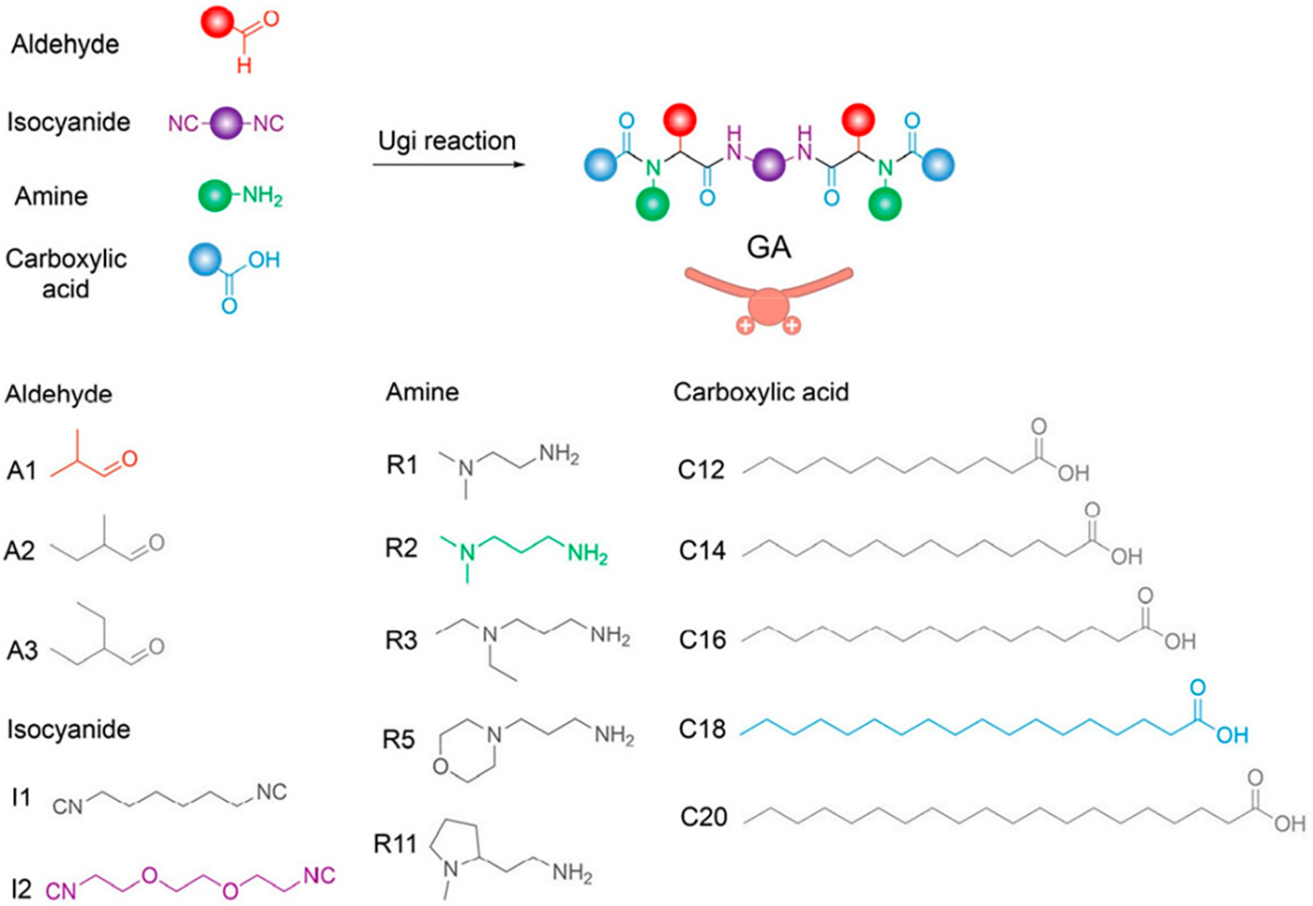
Combinational synthesis of gemini amphiphiles (GAs) for intracellular protein delivery. Incorporation of aldehydes (A), diisocyanides (I), amines (R), and carboxylic acids (C) into GAs via a one-step Ugi multicomponent reaction and layout of the combinational approach. Reproduced with permission from ref (1). Copyright 2023 The Authors. Published by American Chemical Society.

In this work, the authors first synthesized a combinatorial library composed of 150 molecular weight-defined GAs via an isocyanide-mediated Ugi four-component reaction strategy. To understand how a delicate structure impacts its cytosolic delivery performance, they systematically varied each component of the GAs, including aldehydes, diisocyanides, amines, and carboxylic acids, and evaluated the structure–activity relationships. The synthetic GAs in the library were notated as AaIbRxCy, where A*a*, I*b*, R*x*, and C*y* represent the reacted aldehydes (*a* = 1, 2, and 3), diisocyanides (*b* = 1 and 2), amines (*x* = 1, 2, 3, 5, and 11), and carboxylic acids (*y* = 12, 14, 16, 18, and 20), respectively (Figure 1). Their capability for delivering fluorescein isothiocyanate (FITC)-labeled bovine serum albumin (BSA-FITC) into the cytoplasm of HeLa cells was assessed by flow cytometry. The authors found that the GAs synthesized using isobutyraldehyde (A1, red, Figure 1) and *N*,*N*-dimethyl-1,3-propanediamine (R2, green, Figure 1) showed a significantly high cytosolic delivery efficiency. In addition, increases in the alkyl chain length of carboxylic acids from C12 to C20 also resulted in increased cytosolic delivery efficiency, where the trend reached a plateau as the chain length increased to C18 (blue, Figure 1). Longer alkyl chains also greatly improved the cytocompatibility of GAs. Among them, A1I2R2C18 was the most efficient formulation for intracellularly delivering proteins, with FITC-positive rates of about 94%. This finding also revealed that hydrophilic diisocyanides containing ether bonds (I2, purple, Figure 1) were the optimal space linker structure compared to hydrophobic alkyl chains.

**Figure 2:**
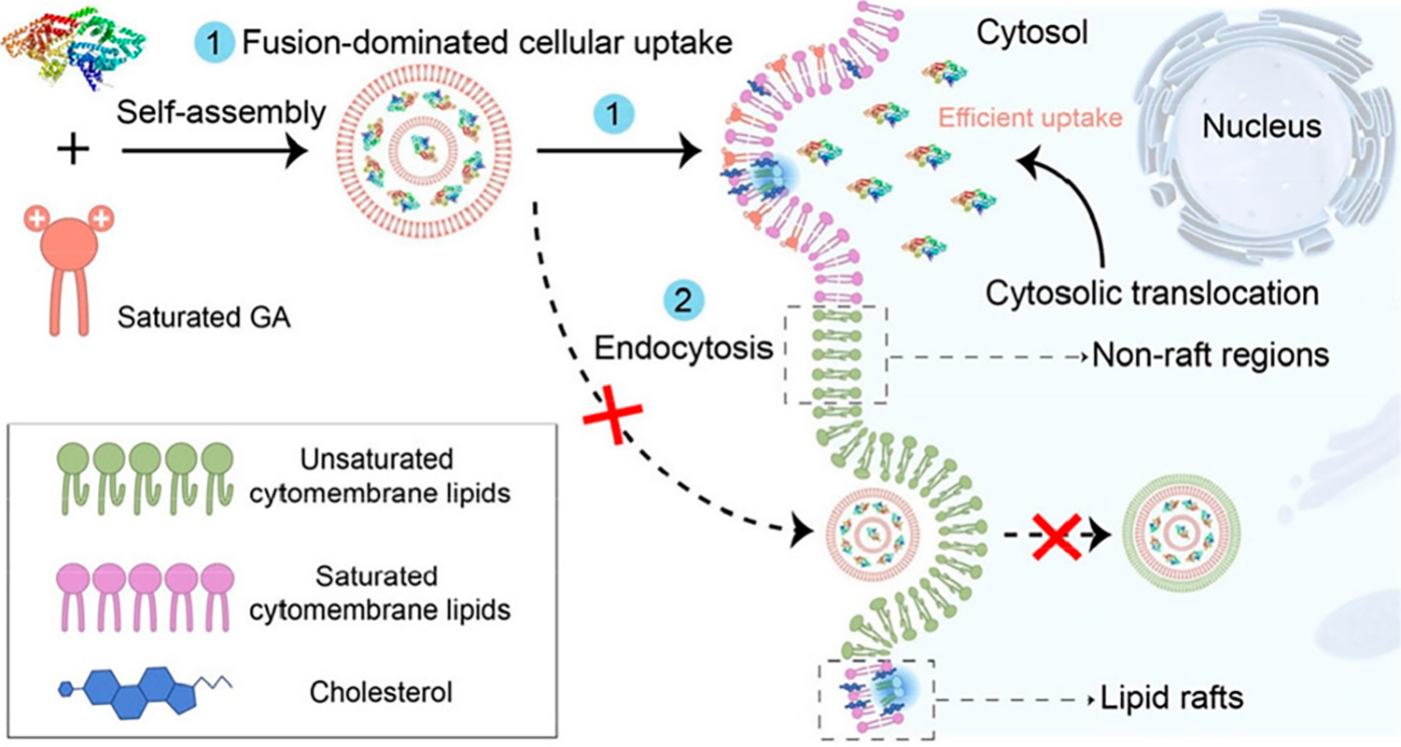
Illustration of the proposed GA-mediated intracellular delivery mechanism. On incubation with cells, the preferential interactions between saturated hydrophobic tails of GAs and lipid raft domains would improve the direct cytosolic delivery of biomacromolecules via the lipid raft-dependent membrane fusion mechanism, thus bypassing the classical endocytic pathway. Reproduced with permission from ref (1). Copyright 2023 The Authors. Published by American Chemical Society.

Then, the authors investigated which cellular internalization mechanism mediated GA uptake *in vitro*. They found that amiloride (the micropinocytosis inhibitor), chlorpromazine (the clathrin-mediated endocytosis inhibitor), and sodium azide (NaN_3_, the energy-dependent endocytosis inhibitor) did not affect A1I2R2C18-mediated cellular uptake behaviors.^2,3^ Moreover, little change in the membrane permeability of endo-/lysosomes was observed during the process of A1I2R2C18-mediated intracellular protein delivery, which was confirmed by calcein and acridine orange staining assays. These results suggested that the cellular internalization mechanism mediating GA uptake was entirely distinct from classical endocytos and bypassed the endo-/lysosomal escape pathway. Next, the authors found that methyl-β-cyclodextrin (MβCD) dramatically inhibited most cellular uptake of A1I2R2C18. MβCD has been reported to scavenge cholesterol through host–guest interactions.^4^ Lipid rafts are cholesterol-enriched heterogeneous and dynamic domains, in which cholesterol has preferential interactions with the saturated lipids. Thus, depletion of membrane cholesterol by MβCD would reduce the affinities between lipid rafts and GAs with saturated alkyl tails.^5,6^ This means that the GA-mediated internalization mechanism should be dependent on lipid raft-mediated translocation pathways (Figure 2). That is, although GAs are structurally similar to cationic lipids, their internalization mechanism is completely different from lipid nanoparticles (LNPs) that mainly involve endocytic pathways.^7^

As proteins are prone to be degraded by proteolysis in endo-/lysosomes, bypassing the endocytic pathway and directly transporting proteins into cells would greatly promote delivery efficiency and enhance therapeutic results. In addition, compared to LNP delivery systems prepared by multicomponent formulations, the authors are excited to find that a single-component GA system is enough to mediate cytosolic delivery of various biomacromolecules for therapeutic purposes. In this work, the successful delivery of Cas9 ribonucleoprotein complex in a tumor-bearing mouse model shows the great potential of GA-based vehicles in biotechnology and biomedicine. The recent success of two mRNA vaccines produced by Pfizer/BioNTech and Moderna for preventing coronavirus 2019 (COVID-19) highlights the enormous potential of LNP-based therapies.^8,9^ Looking forward, in view of the structural similarity to cationic lipids, the potential of GA-based vehicles in mRNA delivery should also be carefully investigated, which would have great clinical and translational values. Also, understanding the influence of surface functionalization on cell-type specific uptake and their safety, and biodistribution *in vivo* would further broaden the application scope of GA-based formulations.^10^
